# Context-dependent associations between heterozygosity and immune variation in a wild carnivore

**DOI:** 10.1186/s12862-015-0519-6

**Published:** 2015-11-04

**Authors:** Patrick M. Brock, Simon J. Goodman, Ailsa J. Hall, Marilyn Cruz, Karina Acevedo-Whitehouse

**Affiliations:** Institute of Biodiversity, Animal Health and Comparative Medicine, College of Medical, Veterinary and Life Sciences, University of Glasgow, Glasgow, G61 1QH UK; Zoological Society of London, Regent’s Park, London, NW1 4RY UK; School of Biology, University of Leeds, Leeds, LS2 9JT UK; Sea Mammal Research Unit, Scottish Ocean Institute, University of St. Andrews, Fife, KY16 8LB UK; Galapagos Genetics, Epidemiology and Pathology Laboratory, Galapagos National Park & University of Guayaquil, Puerto Ayora, Galapagos Islands Ecuador; Unit for Basic and Applied Microbiology, School of Natural Sciences, Autonomous University of Queretaro, Queretaro, 76230 Mexico

**Keywords:** Genetics, Microsatellite, Inbreeding, Immunity, Immunoglobulin, Leukocyte, Life history, Pinniped, *Zalophus wollebaeki*, Galapagos

## Abstract

**Background:**

A multitude of correlations between heterozygosity and fitness proxies associated with disease have been reported from wild populations, but the genetic basis of these associations is unresolved. We used a longitudinal dataset on wild Galapagos sea lions (*Zalophus wollebaeki*) to develop a relatively new perspective on this problem, by testing for associations between heterozygosity and immune variation across age classes and between ecological contexts.

**Results:**

Homozygosity by locus was negatively correlated with serum immunoglobulin G production in pups (0–3 months of age), suggesting that reduced genetic diversity has a detrimental influence on the early development of immune defence in the Galapagos sea lion. In addition, homozygosity by locus was positively correlated with total circulating leukocyte concentration in juveniles (6–24 months of age), but only in a colony subject to the anthropogenic environmental impacts of development, pollution and introduced species, which suggests that reduced genetic diversity influences mature immune system activity in circumstances of high antigen exposure.

**Conclusions:**

These findings demonstrate the environmental context-dependency of the phenotypic expression of immune variation, which is implicit in the theory of ecoimmunology, but which has been rarely demonstrated in the wild. They also indicate that heterozygosity may be linked to the maintenance of heterogeneity in mammalian immune system development and response to infection, adding to the body of evidence on the nature of the mechanistic link between heterozygosity and fitness.

**Electronic supplementary material:**

The online version of this article (doi:10.1186/s12862-015-0519-6) contains supplementary material, which is available to authorized users.

## Background

Associations between indices of multi-locus heterozygosity and many fitness-related traits, from birth weight [1] to song complexity [2], have been reported in wild animal populations [3]. Heterozygosity is of interest because it quantifies within individual genetic diversity, and is likely to be related to inbreeding in some way – although in exactly which way is contentious. Inbreeding has been known to have deleterious effects on fitness for a long time [4, 5], and these effects have been well documented in captive, laboratory and domesticated animals [6]. Historically, the occurrence of inbreeding depression in natural populations was disputed, as evidence was scarcer from the wild than other contexts. It is now thought that this is likely to have been due to detection difficulties [7], and that inbreeding can affect fitness in natural settings [8–11]. The consequences of inbreeding in the wild are of particular interest to biologists working to conserve declining populations or threatened species [9, 10, 12]. Reduced genetic diversity may interact with extrinsic stressors, such as disease, to influence population dynamics [12–15]. Moreover, the extent to which the effects of reduced genetic diversity vary across ecological contexts within species has been relatively poorly explored.

An obstacle to the study of inbreeding in the wild is that there is no problem-free way to estimate levels of inbreeding in a population about which we have incomplete information. For example, the estimation of inbreeding coefficients from pedigrees is complicated by the assumption that founding individuals are outbred and unrelated [16], by the difficulty of accounting for the chance events of Mendelian segregation [17], and by the rarity of multi-generation pedigrees for natural populations [18]. An alternative to calculation from pedigrees is to use data from neutral genetic markers to either summarise genetic diversity as multi-locus heterozygosity (MLH), or to estimate inbreeding coefficients directly using relatedness algorithms, as a proxy for inbreeding value. These latter methods have the advantage of being calculable for individuals sampled from a single cohort, and are therefore feasible in many study systems, especially given recent advances in sequencing technology and the availability of resources for the study of genetics.

However, whether indices of MLH calculated using small numbers of loci represent variation across the genome well enough for inbreeding depression to be invoked as the cause of heterozygosity-fitness correlations (HFCs) is unresolved [19–21]. Empirical comparisons in populations for which both pedigrees and marker data are available have shown that pedigree-derived inbreeding estimates and indices of MLH are not well correlated, especially when the mean and variance of inbreeding value are low [22–26]. However, whether this suggests that MLH indices are less accurate than pedigree-derived estimates or that both are inaccurate in different ways is unknown [17].

An alternative explanation of HFCs to inbreeding depression is that of linkage between one or a few neutral markers and functional genes under balancing selection, which could give rise to the frequently observed pattern of heterosis [19, 27–30]. A reappraisal noted, however, that the uneven contribution of loci to HFCs is implicit in inbreeding theory [16], as weak inbreeding is not expected to lead to detectable identity disequilibrium [16, 31]. Therefore, the effects of weak inbreeding may be present even if heterozygosity is not correlated across loci – an observation that has previously been used to rule out the presence of inbreeding – and the finding that HFCs are driven by variation at one or a few loci does not preclude weak inbreeding as their underlying cause. In addition, the frequentist statistical tests that have been used to identify single-locus contributions to HFCs suffer from problems of power [30] and non-independence [16].

To date, variation in the detection and strength of HFCs has largely been attributed to methodology, and there is consensus that increases in numbers of samples and markers will help delineate the mechanisms that give rise to HFCs [3, 19]. Another way in which HFC studies are working towards this same goal is through the inclusion of ecological heterogeneity in their study designs [3]. This approach co-opts natural variation in the expression of the consequences of inbreeding, for example, through episodic heterozygote advantage [32] or the effects of environmental stress [33, 34], to further understanding of the mechanisms that drive them in natural settings. This study takes such an approach, by examining the relationships between heterozygosity, growth, body condition and immune variation in the Galapagos sea lion.

The Galapagos sea lion (*Zalophus wollebaeki*) is endemic to the Galapagos archipelago and has an estimated population size of 20,000–40,000 animals [35], which is spatially and genetically structured amongst small colonies (20–500 animals) [36, 37]. It has a polygynous mating system [38, 39] and is philopatric [80]. Under these conditions we might expect the inevitable imprecision of marker-based inbreeding estimates to be minimised, as these are both traits that limit the degree to which alleles are mixed within populations, and may therefore promote the necessary variation in heterozygosity required for the statistical detection of underlying patterns [16, 19, 24].

Growth and condition have been empirically linked with survival probability and reproductive success in many taxa [40], including marine mammals [41, 42]. These are likely to be important components of fitness in the Galapagos sea lion as they are related to reproduction, but also because they are related to the ability to resist starvation, which is important in an ecosystem in which fluctuations in marine productivity are driven by unpredictable environmental variation [43, 44]. The concentration of the most common class of circulating mammalian antibody, Immunoglobulin G (IgG), has been linked to survival probability in the grey seal (*Halichoerus grypus*) [42], and the capacity to regulate immunity effectively may be an important determinant of Galapagos sea lion fitness given disease risk concerns [35, 36, 45]. In addition, a recent study reported a link between diversity at an immune gene locus (MHC-DRB) and fitness in the Galapagos sea lion [46]. The data presented here complement this immunogenetic approach, through the inclusion of phenotypic variation in immunity.

The many influences on immune activity in natural populations mean that phenotypic measures of immune variation are unlikely to be related to fitness in straightforward ways [47]. In this study we make use of a previously described longitudinal dataset and sample archive on immune variation in the Galapagos sea lion [48, 49] to take within individual variation, and covariation with other aspects of life history, into account. This previous work describes immune variation in the context of growth and development over the first two years of life in the Galapagos sea lion [48, 49]. Here we build on this by testing for relationships between heterozygosity and immune activity while controlling for the potentially confounding influences of growth, body condition and age class in two breeding colonies, one located in a town and subject to high levels of anthropogenic environmental impact, and the other in a relatively undisturbed habitat [48, 49].

## Methods

### Genotyping

We extracted genomic DNA from 166 Galapagos sea lion skin samples and amplified 23 polymorphic microsatellite loci previously developed for the Galapagos sea lion and other pinniped species [39, 67]. All genetic analyses were carried out at the University of Bielefeld, Germany, as described in [39, 67]. Sequencing was performed on an Applied Biosystems 3130 Sequencer (Life Technologies) and genotyping was performed in GENEMARKER (Soft Genetics, USA). We tested for deviations from Hardy-Weinberg equilibrium (HWE) using Monte Carlo simulations in the *adegenet* package [68] in R 2.14.1 [69], and for identity disequilibrium using the *g*_2_ measure [31].

### Main statistical analysis

Samples were collected in two Galapagos sea lion colonies: one on an uninhabited island (control colony, Santa Fe), and one located in a town (human-impacted colony, San Cristobal) [48]; and from sea lions in two ecologically distinct age-classes: pups (0–3 months old), which are restricted to land and are dependent on their mothers for nutrition; and juveniles (6–24 months old), which swim out to sea and are capable of foraging independently [70]. We sampled pups shortly after birth and at 3 months of age, between which two ages they undergo a growth spurt [44]; and juveniles at 6, 12, 18 and 24 months of age. We captured sea lions using hoop nets and briefly restrained them in a prone position without the use of chemical immobilization, and without causing harm, by following the capture protocol in [70]. All work was approved by the Zoological Society of London Ethics Committee, and carried out under Galapagos National Park permits PC-18-09, N046-2009-PNG, N101-2010-PNG and N032-2010-PNG, which covered all fieldwork, capture and sample protocols. We used two measures of immune variation: immunoglobulin G (IgG) concentration and total leukocyte concentration, as they were highlighted in previous analyses as most likely to vary meaningfully with other aspects of Galapagos sea lion life history [48, 49].

Galapagos sea lion pups undergo rapid growth and physiological development during the sampled period of their early development [44]. In order to take these changes into account, and given that pups were only sampled at two time points during this period, we calculated absolute changes in body mass (kg), body length (cm), IgG concentration (mg/ml) and total leukocyte concentration (10^9^/l) between shortly after birth and 3 months of age. The possibility of phenotypic correlation [54] means that growth and changes in immune measures may covary, and we have shown that the direction of these associations varies between colonies in the Galapagos sea lion [49]. Therefore, in fast-growing pups, we partitioned variation in changes in each immune measure into subsets that were correlated with changes in body length and body mass in different ways using principal components analyses, carried out separately for each colony. For each colony and immune measure we fit generalised linear models with principal components that explained ≥ 5 % of the variation as response variables to homozygosity weighted by locus (HL) [52], sex and their interaction as explanatory terms, removing interactions if they were non-significant [71]. This amounted to eight statistical models fitted to pup data: two principal components, from two immune measures, in two colonies. This approach addresses the problem of the potentially confounding influence of phenotypic correlation on associations between HL and changes in immune measures in fast-growing pups, as it partitions the variation in changes in immune measures into components that are correlated with different kinds of growth, and allows for comparison between their association with homozygosity.

We chose HL as the most appropriate measure of heterozygosity for the main statistical analyses, so that they could be compared with other published results (e.g. [46]), and because the distribution of the variation in HL was amenable to modelling in a generalised linear model (GLM) framework. However, we undertook a detailed exploration of the biases inherent in different estimates of heterozygosity and inbreeding using simulation analysis to provide context for these results, and other analyses that use measures of heterozygosity more generally (Additional file 1: Supplementary Text 1.1–2, Table S2–3, Figure S1).

In comparison with pups, relatively little growth occurs in juvenile Galapagos sea lions between the ages of 6 and 24 months [44], fewer physiological changes take place, and body mass and length are more closely correlated than in younger animals [49]. In addition, we sampled juveniles at four rather than two time points. The nature of the juvenile data, therefore, allowed us to take a simpler approach to correcting for phenotypic correlation, which we did by including body mass as an explanatory variable. Separately for each colony, we fitted generalised linear mixed models (GLMMs) with each immune measure as a response variable and HL, body mass, sex and the interaction between HL and sex as explanatory terms. We included individual identity as a random effect to account for the pseudoreplication implicit in the repeated sampling of individuals. This amounted to four statistical models fitted to juvenile data, which covered two colonies and two immune measures. We compared models with and without the interaction between sex and HL using likelihood ratios tests [72]. The analysis of juvenile data was therefore analogous to that of pup data, but did not require partitioning by principal components analysis. We checked all models for signs of heteroscedasticity, heterogeneity of variance, non-normality of error and the disproportionate influence of outliers.

## Results

Of 23 amplified loci, one (ZcwG06) [50] showed significant deviation from Hardy-Weinberg equilibrium (Additional file 1: Table S1) and was omitted from all analyses. All individuals were genotyped twice, samples that produced allele mismatches were excluded from the analyses (*n* = 2), and the data are provided in Additional file 2. Homozoygosity by locus (HL) did not vary significantly with sex (GLM: *n* = 52, estimate = −0.50, standard error, SE = 0.42, *p* = 0.23), colony (GLM: n = 52, estimate = −0.52, SE = 1.57, *p* = 0.74) or estimated birthdate (GLM: *n* = 52, estimate = 0.012, SE = 0.045, *p* = 0.78) in pups; or with sex (GLM: *n* = 73, estimate = 0.03, SE = 0.02, *p* = 0.137) or colony (GLM: *n* = 73, estimate = −0.03, SE = 0.02, *p* = 0.09) in juveniles. There was no evidence of identity disequilibrium (*g*_2_ = 0.0033, standard deviation, SD = 0.0027, *p* < 0.888, 1000 iterations).

The mean change in pup body mass from shortly after birth until 3 months of age was 6.01 kg (SD = 1.81 kg) and mean change in pup body length over the same period was 14.59 cm (SD = 5.19 cm). Change in pup body length was greater in the human-impacted colony than in the control colony (GLM: *n* = 40, estimate = −3.93, SE = 1.54, *p* = 0.014), but there was no significant difference between sexes (GLM: *n* = 40, estimate = 3.14, SE = 1.59, *p* = 0.056). Change in body mass was greater in male pups than female pups (GLM: *n* = 40, estimate = 1.39, SE = 0.54, *p* = 0.013), but there was no significant difference between colonies (GLM: *n* = 40, estimate = −0.99, SE = 0.56, *p* = 0.083).

For IgG, the first component produced by principal component analyses for each colony was positively correlated with changes in IgG concentration, changes in body mass, and changes in body length (0.93, 0.33, 0.09 and 0.92, 0.33, 0.15 in the human-impacted and control colonies respectively; Table 1); and the second component was positively correlated with changes in IgG concentration but negatively correlated with changes in body mass and length (0.34, −0.83, −0.43 and 0.36, −0.91, −0.17 in the human-impacted and control colonies respectively; Table 1). This demonstrates the separation of variation in IgG into that which is positively correlated with growth through phenotypic correlation, represented by the first principal components, and that which is not, represented by the second principal components. For total leukocyte concentration, the principal component analyses produced a similar pattern to IgG, and for both IgG and total leukocyte concentration, the differences between colonies were minimal (Table 1; Additional file 1: Figure S2).

**Table 1:** Principal component analysis results for IgG (total immunoglobulin G concentration, mg/ml) in the human-impacted

a) Human-impacted with IgG	Component 1	Component 2	Component 3
Δ IgG (mg/ml)	0.936	0.344	0.062
Δ Length (cm)	0.335	−0.834	−0.437
Δ Mass (kg)	0.098	−0.430	0.897
Standard deviation	12.361	3.132	1.013
Proportion of variance	0.933	0.059	0.006
Cumulative proportion of variance	0.933	0.993	1
b) Control with IgG	Component 1	Component 2	Component 3
Δ IgG (mg/ml)	0.928	0.362	−0.083
Δ Length (cm)	0.337	−0.916	−0.215
Δ Mass (kg)	0.154	−0.171	0.973
Standard deviation	10.907	4.785	1.133
Proportion of variance	0.831	0.160	0.009
Cumulative proportion of variance	0.831	0.991	1
c) Human-impacted with WBC	Component 1	Component 2	Component 3
Δ WBC (10^9^/l)	−0.328	0.936	−0.123
Δ Length (cm)	−0.877	−0.351	−0.326
Δ Mass (kg)	−0.349	0.001	0.937
Standard deviation	4.944	2.748	1.095
Proportion of variance	0.736	0.227	0.036
Cumulative proportion of variance	0.736	0.963	1
d) Control with WBC	Component 1	Component 2	Component 3
Δ WBC (10^9^/l)	0.102	0.964	−0.243
Δ Length (cm)	−0.957	0.029	−0.287
Δ Mass (kg)	−0.269	0.262	0.926
Standard deviation	6.002	2.518	1.243
Proportion of variance	0.820	0.144	0.035
Cumulative proportion of variance	0.820	0.964	1

(a) and control (b) colonies, and WBC (total leukocyte concentration, 109/l) in the human-impacted (c) and control (d) colonies, with growth variables in pups. Δ denotes ‘change in’

**Fig. 1:**
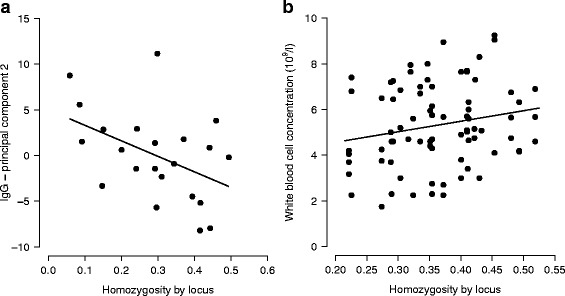
**a** The relationship between homozygosity weighted by locus (HL) and the second principal component derived from pup data on changes in body length, mass and IgG concentration in the control colony (GLM: estimate = 16.75, SE = 7.58, *p* = 0.039; Tables 2 and 3); (**b**) the relationship between homozygosity weighted by locus (HL) and total leukocyte concentration (10^9^/l) in juveniles in the human-impacted colony (GLMM: estimate = 5.98, SE = 2.21; LRT: χ^2^ = 7.29, *p* = 0.006; Tables 2 and 3)

In pups homozoygosity by locus (HL) was negatively associated with the second principal component derived from IgG in the control colony (GLM: estimate = 16.75, SE = 7.58, *p* = 0.039; Tables 2 and 3; Fig. 1a). This principal component was most strongly correlated with changes in body length (−0.91), next with changes in IgG (0.36) and lastly with changes in mass (−0.17; Table 1). The pattern of variation observed with HL suggests that individuals with high levels of heterozygosity grew less but produced more IgG. Sex was included in the statistical models to account for its potentially confounding influence but was never significant as part of an interaction with HL. In juveniles the interaction between HL and sex was not retained in any model, and HL was retained in a single model, in which it was positively correlated with total leukocyte concentration in the human-impacted colony (GLMM: estimate = 5.98, SE = 2.21; likelihood ratio test, LRT: χ^2^ = 7.29, *p* = 0.006; Tables 2 and 3 and Fig. 1b).

**Table 2:** Statistical associations between homozygosity weighted by locus (HL) and principal components derived from immune and growth variables and in pups (linear models), and immune variables in juveniles (linear mixed effect models)

Colony	Response Variable	Estimate (HL)	SE	*t*-value	*p*-value
HIC	PC1-IgG	0.60	45.55	0.01	0.989
	PC2-IgG	−13.28	11.55	−1.15	0.266
CC	PC1-IgG	27.99	18.48	1.51	0.146
	PC2-IgG	−16.75	7.58	−2.20	0.039 *
HIC	PC1-WBC	−21.32	17.47	−1.22	0.240
	PC2-WBC	10.87	10.28	1.05	0.306
CC	PC1-WBC	−16.23	9.12	−1.77	0.089
	PC2-WBC	2.01	3.665	0.55	0.588

*HIC* human-impacted colony, *CC* control colony, *SE* standard error, *IgG* total immunoglobulin G concentration (mg/ml), *WBC* total leukocyte concentration (10^9^/l), *p<0.05

**Table 3:** Statistical associations between homozygosity weighted by locus (HL) and principal components derived from immune and growth variables and immune variables in juveniles (linear mixed effect models)

Colony	Response Variable	Chi-squared	*p*-value	Estimate	SE	*t*-value
HIC	WBC	7.299	0.006 **	5.985	2.215	2.701
	IgG	0.009	0.864	-	-	-
CC	WBC	0.651	0.419	-	-	-
	IgG	3.264	0.071	-	-	-

In juveniles the Chi-squared and *p*-values are for likelihood ratio tests that compared models with HL, sex and mass as explanatory variables with models including only sex and mass; the estimate, standard error and *t*-values are for HL and are only reported for models in which HL was retained

*HIC* human-impacted colony, *CC* control colony, *SE* standard error, *IgG* total immunoglobulin G concentration (mg/ml), *WBC* total leukocyte concentration (10^9^/l). **p<0.01

## Discussion

This study has two main findings: first, two different kinds of immune variation were associated with heterozygosity in a wild mammal; second, the occurrence and nature of these associations varied between ecologically distinct circumstances. The latter allows for the possibility that human impact may influence the relationship between genotype and immune phenotype in this species [53]. These results – as is necessarily the case with studies of species such as the Galapagos sea lion in the wild – are correlative, and preclude the inference of direct causation. Also, given that the colony on San Cristobal (human-impacted) is unique – as discussed in detail in [48, 49] – it was not possible to test for interactions between heterozygosity and human-impact on immune variation across a larger sample of colonies. Nevertheless, the results reported here answer a call to incorporate more ecologically relevant variation into the exploration of the impacts of variation in heterozygosity in natural populations [3]. In addition, given recent interest in the relationship between variation at immune gene loci and fitness in wild organisms, including in the Galapagos sea lion [46], the quantification of physiological variation in immunity may prove a valuable tool for understanding the complex mechanisms that give rise to correlations between such immunogenetic variation and fitness.

### Immunoglobulin G (IgG) and heterozygosity

Physiological measures of immune variation may be condition-dependent [54], and variation in IgG concentration is correlated with growth in Galapagos sea lion pups [49]. We partitioned variation in growth and IgG production into principal components to take this co-linearity into account. The majority of Galapagos sea lion pups grow [44] and produce IgG [48] during their first three months of life. Given the lack of evidence for infection or disease in the sampled Galapagos sea lion pups (Additional file 1: Supplementary text 1.3–1.4), and a field experiment that showed IgG is not passed from mother to pup during the sampled stage of development in the Galapagos sea lion [48], the observed IgG production is likely to be driven by post-natal antigen exposure, which stimulates the build-up of protective baseline populations of lymphocytes and antibodies [42, 55].

Homozygosity weighted by locus (HL) was not associated with the first principal component derived from changes in IgG concentration, body mass and body length. This suggests that heterozygosity may not be straightforwardly related to fitness, as it was not correlated with the axis of variation that most likely describes phenotypic correlation between traits (Tables 2 and 3) [54]. The second principal component was negatively correlated with HL, which, given the loadings (Tables 2 and 3), suggests that relatively heterozygous individuals grew less but produced more IgG, which is congruent with findings from other systems [14, 56]. Such a pattern could arise through variation in resource allocation or acquisition between sea lion pups with different levels of heterozygosity [57]. However, this does not explain why relatively homozygous pups appeared to grow more. The answer to this may lie in the relative magnitude of the length and mass loadings on the two components: there is a small difference between these in the first component (0.33 and 0.15 for length and mass respectively; Tables 2 and 3), as we would expect under phenotypic correlation; but length loads more heavily on the second component than mass (−0.91 and −0.17 for length and mass respectively; Tables 2 and 3). This suggests that the first component better represents overall growth that is more likely to be positively correlated with fitness, while the second represents elongation, which corresponds to a decrease in body condition [40].

Pups from the human-impacted colony varied less in terms of heterozygosity than pups from the control colony (Additional file 1: Figure S1a-b), so the power to detect an effect may have been lower in the former, which could explain why the above pattern was only observed in the control colony. An alternative explanation is that pups (or mothers) in the human-impacted colony did not vary resource allocation according to heterozygosity in the same way that those in the control colony did. Pups in the human-impacted colony produced more IgG than pups from the control colony during the sampled period of development, which may be due to higher post-natal antigen exposure in the human-impacted colony [48]. If this is the case, environmental stimulation of IgG production in the human-impacted colony may have over-ridden genetic influence on IgG production, which could obscure the statistical signal of genetic influence on IgG production in the human-impacted colony.

### Leukocytes and heterozygosity

Total leukocyte concentration in mammals is likely to vary over shorter timescales than IgG concentration, and to be more prone to fluctuation, as leukocytes are less cumulative in the blood than antibodies [58]. In young mammals, though, (including sea lions) total leukocyte concentration changes with age, as cell populations develop during post-natal growth [48, 59]. Therefore, in pups we used the same principal component analysis approach as we did with IgG. However, heterozygosity was not statistically associated with any of the principal components derived from total leukocyte concentration in pups (Table 1).

The positive correlation between homozygosity and total leukocyte concentration in juveniles in the human-impacted colony suggests that low within individual genetic diversity may be associated with an increase in the number of circulating leukocytes, under certain ecological circumstances. Total leukocyte concentration in juveniles is more likely to represent mature immune system activity than early-developmental leukocyte production, given that the latter happens during only a short period following birth in pinnipeds [60, 61]. Low heterozygosity has been associated with increased susceptibility to parasitism [1, 13, 62], so may have compromised aspects of innate immunity, e.g. [14, 56], in these juvenile sea lions, which could have led to relatively high infection rates, e.g. [63]. This may have been observed only in the human-impacted colony as this is where infection risk is likely to be relatively high, given the threat of pathogen exposure [36, 45, 52]; an effect that may be compounded by relatively high stress and pollution levels in the human-impacted colony [48].

### Heterozygosity and inbreeding

A general assumption of heterozygosity-fitness-correlation (HFC) studies is that phenotypic variation is linearly related to fitness and therefore under directional selection [3]. Immune variables such as IgG concentration are unlikely to be so, due to the damage caused by immunopathology and other trade-offs involving immunity [57]; moreover, different kinds of antibody responses have been shown to be under different kinds of selection: in blue tits (*Parus caeruleus*) “…primary responsiveness to diphtheria was subject to stabilizing selection, whereas secondary responsiveness to tetanus was subject to positive directional selection” [47]. Correlations between such traits and heterozygosity are nonetheless useful, as when, and under what ecological circumstances, they are detectable can offer insight into their mechanisms [3]. Immune traits are unlikely to be influenced by as many genes as complex composite traits such as survival and lifetime reproductive success (to which they contribute), and therefore may be more amenable to a candidate gene approach to the investigation of HFC mechanisms, e.g. [64]. However, this also implies that immune traits are less likely to be influenced by genome-wide inbreeding [16], which raises the question of whether heterozygosity is acting as a proxy for inbreeding in the Galapagos sea lion.

As *g*_2_ was not significantly different from zero (*g*_2_ = 0.0033, SD = 0.0027, *p* < 0.888, 1000 iterations), identity disequilibrium of our marker set was low [31]. This suggests an absence of inbreeding (but see [21]) in the Galapagos sea lion, which agrees with other assessments using different proxies for inbreeding in the Galapagos sea lion [37, 46]. If the correlations between heterozygosity and immune variation described here are not due to inbreeding, it is possible they are the result of the other widely suggested explanation for HFCs: linkage between one or a few presumed-neutral markers to functional genes under balancing selection [19]. A variety of statistical approaches have been proposed to identify heterogeneities in the contributions of loci to HFCs [19, 27, 28, 65], but problems of power and non-independence mean their results are difficult to assess [16, 30]. In addition, unbalanced contributions of one or a few loci to HFCs are implicit in inbreeding theory, as weak inbreeding is not expected to lead to detectable identity disequilibrium [16, 31]. Therefore, an absence of identity disequilibrium (or other equivalent measure of correlation of heterozygosity across loci) does not preclude the presence of weak inbreeding [16].

## Conclusions

Although the links between heterozygosity and inbreeding are unresolved [20], the context-dependent expression of patterns of covariance between heterozygosity and other traits can yield useful and interesting information. We offer these results as examples of covariation between aspects of genotype and phenotype in a natural population, which demonstrate the context-dependency of immune variation expression predicted by ecoimmunology, and which may provide new perspective on heterozygosity-fitness correlations, by allowing for the formation of increasingly detailed hypotheses on the mechanisms that drive them. Irrespective of their causes and mechanisms, and in light of recent findings in the Antarctic fur seal (*Arctocephalus gazelle*) [12], our results may also have implications for the management of declining populations, as they allow for the possibility that genetic effects interact with the two major threats to the Galapagos sea lion: climate-driven changes in food availability [35, 44, 66] through effects on growth and condition; and disease threat [36], through effects on immunity; both of which could act to compound the risk of decline and extinction.

### Availability of supporting data

The genetic data used in the analyses are available in Additional file 1.

## Additional files

Additional file 1: Table S1. Tests for deviations from Hardy-Weinberg equilibrium. Supplementary text 1.1: Simulation methods. Supplementary text 1.2: Simulation results. **Tables S2.** & **S3.** Correlations with simulated inbreeding coefficients. **Figure S1.** Triadic likelihood estimator vs. homozygosity weighted by locus. **Figure S2.** Principal component analysis results. Supplementary text 1.3: Parasites methods. Supplementary text 1.4: Parasite results. (DOCX 95 kb) Additional file 2:
**Microsatellite data.** (CSV 30 kb)

## Abbreviations

MLH: Multi-locus heterozygosity
HFC: Heterozygosity-fitness correlations
IgG: Immunoglobulin G
HWE: Hardy-Weinberg equilibrium
HL: Homozygosity weighted by locus
GLM: Generalised linear model
GLMM: Generalised linear mixed model
SE: Standard error
SD: Standard deviation
LRT: Likelihood ratio test
