# Supplementary material for: Context-dependent associations between heterozygosity and immune variation in a wild carnivore
Source: BMC Evol Biol. 2015 Nov 4;15:242. doi: 10.1186/s12862-015-0519-6 (PMC4634738; doi:10.1186/s12862-015-0519-6)
Supplement: Additional file 1: Table S1. — Tests for deviations from Hardy-Weinberg equilibrium. Supplementary text 1.1: Simulation methods. Supplementary text 1.2: Simulation results. Tables S2. & S3. Correlations with simulated inbreeding coefficients. Figure S1. Triadic likelihood estimator vs. homozygosity weighted by locus. Figure S2. Principal component analysis results. Supplementary text 1.3: Parasites methods. Supplementary text 1.4: Parasite results. (DOCX 95 kb) [file 12862_2015_519_MOESM1_ESM.docx]

Brock, Goodman, Hall, Cruz & Acevedo-Whitehouse; *Context-dependent associations between heterozygosity and immune variation in a wild carnivore;* BMC Evolutionary Biology

Additional file 1

Table S1. Tests for deviations from Hardy-Weinberg equilibrium (HWE) for each locus, carried out using Monte Carlo simulations (n = 10,000) in the ‘adegenet’ package (Jombart 2008) in R 2.14.1 (R Development Core Team, 2011).

| Locus | HWE probability test *p*-value |
| --- | --- |
| ZcwA05 | 1 |
| Pv9 | 1 |
| ZcwD01 | 1 |
| Hg4.2 | 0.620 |
| ZcwA12 | 1 |
| ZcwE05 | 0.985 |
| ZcwH09 | 0.790 |
| ZcwD02 | 1 |
| ZcwA07 | 1 |
| ZcwB09 | 1 |
| ZcwC03 | 1 |
| ZcCgDh5.8 | 1 |
| ZcwC11 | 1 |
| Hg8.10 | 0.988 |
| ZcCgDh7tg | 0.999 |
| Hg6.1 | 1 |
| ZcwF07 | 0.988 |
| ZcwE03 | 0.860 |
| ZcwE12 | 0.972 |
| ZcwE04 | 1 |
| Pv11 | 0.174 |
| ZcwG06 | 0.007 ** |
| ZcwB07 | 0.120 |

*Supplementary text 1.1 Simulation methods*

To explore how HL compared with estimates of inbreeding in the context of our dataset, we calculated the triadic maximum likelihood estimator of inbreeding value (TML) [51] for each individual using the empirical allele frequencies from each colony (human-impacted colony n = 71, control colony = 95), and compared these with HL. To further compare the utility of different marker-based estimates of heterozygosity and inbreeding as proxies for true inbreeding value in the context of our dataset, we performed simulations in COANCESTRY [73]. We used allele frequencies from the data to simulate four populations of known relatedness structure and compared the resulting values of the inbreeding coefficient (*f*) with neutral marker-based inbreeding estimates and MLH indices calculated from the simulated genotypes. We calculated two moment estimators [74-75] and two likelihood estimators of inbreeding [51,76] in COANCESTRY [73]. In addition, we calculated three indices of MLH: standardised heterozygosity (SH) [63]; internal relatedness (IR) [77]; and homozygosity weighted by locus (HL) [52] in the Rhh package [78] in R 2.14.1 [69]. An advantage of the COANCESTRY [73] likelihood framework is that it takes into account genotyping error rates [39].

Following [10] we simulated the first population with a uniform distribution of *f*, in which *f* increased in increments of 0.05 from 0 to 1, and each category included 100 individuals (n = 2,100). We simulated the second population with the same uniform distribution but limiting the range of *f* from 0 to 0.5 and increasing the number of individuals in each category to 200 so that the population sizes were similar (n = 2,200). We limited the range in this way after estimating inbreeding values from the data, and observing that estimators on the same scale as *f* were always below 0.5. Next, we used the triadic maximum likelihood estimator of inbreeding value (TML) [51] to estimate the empirical distribution of inbreeding values from the data. Then we simulated the third population using this distribution, adjusting the number of individuals in each inbreeding value category to match the total number of individuals in the first two simulated populations as closely as possible (n = 2,088). We simulated the fourth population using the empirical distribution of TML inbreeding value estimates and the empirical sample size (n = 166). Finally, we correlated the four inbreeding estimators and three MLH indices with the value of *f* in each simulated population.

*Supplementary text 1.2 Simulation results*

In all four populations simulated to compare different indices of heterozygosity and estimates of inbreeding value correlation with true inbreeding value (*f*) was higher for likelihood methods than it was for either moment estimators or MLH indices (Table S2). While MLH indices performed better in simulated populations with uniform distributions of *f*, moment estimators performed better when populations were simulated with more realistic, right-tailed *f* distributions (Table S2). Despite the advantages of the likelihood estimators highlighted by these simulation analyses, we found that fitting statistical models with triadic likelihood estimator of inbreeding (TML) [51] as an explanatory variable led to worrisome heteroscedasticity and unstable parameter estimates. Therefore, we made a detailed visual comparison of TML with homozygosity weighted by locus (HL) [52] (Figure S1), and conducted all statistical analyses using HL.

Table S2. Mean correlations between simulated values of the inbreeding coefficient *f* and indices of multi-locus heterozygosity (MLH), moment estimators of inbreeding and likelihood estimators of inbreeding in four simulated populations. MLH indices were standardized heterozygosity (SH) [63], internal relatedness (IR) [77] and homozygosity weighted by locus (HL) [52]; moment estimators were Ritland [74] and Lynch and Ritland [75]; likelihood estimators were dyadic [76] and triadic methods [51]. “Uniform” distributions had an equal number of individuals in each inbreeding value category, while in “GSL” distributions the number of individuals in each inbreeding value category was proportional to empirical estimates from Galapagos sea lion data. See Table S2 for more detail.

|  |  |  |  | Mean correlation with *f* | | |
| --- | --- | --- | --- | --- | --- | --- |
|  | Distribution | Range of *f* | Number of individuals | Heterozygosity indices | Moment estimators | Likelihood estimators |
|  | Uniform | 0 – 1.0 | 2,100 | 0.917 | 0.794 | 0.943 |
|  | Uniform | 0 – 0.5 | 2,200 | 0.726 | 0.618 | 0.767 |
|  | GSL | 0 – 0.5 | 2,088 | 0.416 | 0.437 | 0.512 |
|  | GSL | 0 – 0.5 | 166 | 0.326 | 0.388 | 0.404 |

Table S3. Correlations between simulated values of *f*, 4 inbreeding estimators and 3 indices of multi-locus heterozygosity (MLH), for different simulated distributions and ranges of *f*, and different sample sizes: (a) uniform distribution of *f* from 0 to 1 in steps of 0.05 with 100 individuals in each category (n = 2,100); (b) uniform distribution of *f* from 0 to 0.5 in steps of 0.05 with 200 individuals in each category (n=2,200); (c) simulated distribution of *f* modelled on empirical distribution estimated from Galapagos sea lion data, from 0 to 0.5 in steps of 0.05, (n = 2,088); (d) simulated distribution of *f* modelled on the empirical distribution estimated from Galapagos sea lion data from 0 to 0.5 in steps of 0.05, empirical sample size (n=166). MLH indices were standardized heterozygosity (SH) [63], internal relatedness (IR) [77] and homozygosity weighted by locus (HL) [52]; moment estimators were Ritland [74] and Lynch and Ritland [75]; likelihood estimators were dyadic [76] and triadic methods [51].

| (a) | True Value | RIT | LYRT | DML | TML | SH | IR |
| --- | --- | --- | --- | --- | --- | --- | --- |
| RIT | 0.675 |  |  |  |  |  |  |
| LYRT | 0.913 | 0.784 |  |  |  |  |  |
| DML | 0.944 | 0.702 | 0.955 |  |  |  |  |
| TML | 0.942 | 0.704 | 0.957 | 0.998 |  |  |  |
| SH | 0.914 | 0.677 | 0.929 | 0.968 | 0.972 |  |  |
| IR | 0.922 | 0.688 | 0.938 | 0.976 | 0.979 | 0.994 |  |
| HL | 0.914 | 0.677 | 0.929 | 0.968 | 0.972 | 1.000 | 0.994 |
|  |  |  |  |  |  |  |  |
| (b) | True Value | RIT | LYRT | DML | TML | SH | IR |
| RIT | 0.519 |  |  |  |  |  |  |
| LYRT | 0.718 | 0.811 |  |  |  |  |  |
| DML | 0.764 | 0.594 | 0.857 |  |  |  |  |
| TML | 0.769 | 0.599 | 0.864 | 0.992 |  |  |  |
| SH | 0.720 | 0.521 | 0.784 | 0.907 | 0.919 |  |  |
| IR | 0.738 | 0.555 | 0.811 | 0.929 | 0.940 | 0.982 |  |
| HL | 0.720 | 0.521 | 0.784 | 0.907 | 0.919 | 1.000 | 0.982 |
|  |  |  |  |  |  |  |  |
| (c) | True Value | RIT | LYRT | DML | TML | SH | IR |
| RIT | 0.367 |  |  |  |  |  |  |
| LYRT | 0.409 | 0.941 |  |  |  |  |  |
| DML | 0.408 | 0.719 | 0.776 |  |  |  |  |
| TML | 0.400 | 0.733 | 0.789 | 0.979 |  |  |  |
| SH | 0.326 | 0.631 | 0.684 | 0.796 | 0.811 |  |  |
| IR | 0.327 | 0.657 | 0.709 | 0.826 | 0.838 | 0.969 |  |
| HL | 0.326 | 0.631 | 0.684 | 0.796 | 0.811 | 1.000 | 0.969 |
| (d) | True Value | RIT | LYRT | DML | TML | SH | IR |
| RIT | 0.400 |  |  |  |  |  |  |
| LYRT | 0.473 | 0.850 |  |  |  |  |  |
| DML | 0.518 | 0.586 | 0.786 |  |  |  |  |
| TML | 0.505 | 0.602 | 0.805 | 0.981 |  |  |  |
| SH | 0.407 | 0.476 | 0.665 | 0.784 | 0.805 |  |  |
| IR | 0.434 | 0.524 | 0.713 | 0.822 | 0.841 | 0.966 |  |
| HL | 0.407 | 0.476 | 0.665 | 0.784 | 0.805 | 1.000 | 0.966 |

Figure S1. Comparison between values of triadic likelihood estimator of inbreeding (TML) [51] with homozygosity weighted by locus (HL) [52] estimated from our dataset: a-d) histograms of HL and TML in pups by colony, e) HL plotted against TML in pups, f-i) histograms of HL and TML in juveniles by colony, and j) HL plotted against TML in juveniles; HIC = human-impacted colony, CC = control colony; all values calculated using 22 loci.

Figure S2. Results from the principal components analysis partitioning variation between change in total immunoglobulin G concentration (IgG, mg/ml) in the human-impacted (a) and control (b) colonies, and WBC (total leukocyte concentration, 10^9^/l) in the human-impacted (c) and control (d) colonies. See Table 2 for the full tabulated results.

b)

a)

d)

c)

*Supplementary text 1.3 Parasites methods*

We set out to estimate the infection status and parasite burden of the sampled Galapagos sea lions in three ways. First, we conducted external examinations during each capture to quantify infection with ectoparasites and the trematode *Philophthalmus zalophi* [79]. Second, while carrying out differential white blood cell counts [48] we screened blood smears for blood-borne parasites, particularly microfilariae [79]. Third, we screened faecal samples, which were collected by rectal swabbing during captures and opportunistically when known individuals were observed defecating on the beach, for the presence of hookworm eggs. We examined swab samples using direct smears and beach samples by flotation, using a McMaster chamber to count eggs after centrifugation and saturation of the sample with sugar solution (E. T. Lyons, personal communication).

*Supplementary text 1.4 Parasite results*

We encountered ectoparasites in 15 out of 487 external examinations carried out during sea lion captures. All 15 of these observations were of lice (*Antarctophthirus microchir*), nine findings were of a single louse and the remaining six were of two lice. Lice were most often found on the ventral surface of the abdomen just anterior to the hind flippers. Given the small number of sea lions positive for ectoparasite infection, we did not include this data in the analysis. Eye examinations for the determination of *P. zalophi* infection status were possible during 207 captures; 126 of these individuals were infected and 81 were not. However, the burden of infection varied between infected individuals in a way that could not be reliably quantified. This was partly due to differences in individual behaviour: during many captures examination of the ocular cavity was difficult and incomplete, and in others it was not feasible at all. Therefore, despite the prevalence of this parasite, it was not possible to quantify infection and burden across individuals consistently. Consequently, we also excluded this data from the analysis. We screened 280 blood sears for the presence of blood-borne parasites such as microfilariae, but none showed any signs of such infections. We examined 79 faecal swabs collected during captures and 20 faecal samples collected from the beach. Only one of these beach samples was from a known individual, the others were collected in an attempt to validate the methods. However, no hookworm eggs were detectable in any of the samples.
